# Influence of Gonadotropin Hormone Releasing Hormone Agonists on Interhemispheric Functional Connectivity in Girls With Idiopathic Central Precocious Puberty

**DOI:** 10.3389/fneur.2020.00017

**Published:** 2020-01-31

**Authors:** Tao Chen, Wenquan Yu, Xiaoling Xie, Huaizhi Ge, Yuchuan Fu, Di Yang, Lu Zhou, Xiaozheng Liu, Zhihan Yan

**Affiliations:** ^1^Department of Radiology, The Second Affiliated Hospital and Yuying Children's Hospital of Wenzhou Medical University, Wenzhou, China; ^2^Department of Radiology, Zhejiang Hospital, Hangzhou, China; ^3^Department of Radiology, The Children's Hospital, Zhejiang University School of Medicine, Hangzhou, China

**Keywords:** gonadotropin-releasing hormone agonists, idiopathic central precocious puberty, hypothalamic-pituitary-gonadal (HPG) axis, luteinizing hormone, functional magnetic resonance imaging, voxel-mirrored homotopic connectivity

## Abstract

**Purpose:** The pubertal growth suppressive effects of gonadotropin hormone releasing hormone agonists (GnRHa) are well-known, although it remains unclear if long-term GnRHa treatment influences the brain function of treated children. The present study investigated the differences in the homotopic resting-state functional connectivity patterns in girls with idiopathic central precocious puberty (ICPP) with and without GnRHa treatment using voxel-mirrored homotopic connectivity (VMHC).

**Methods:** Eighteen girls with ICPP who underwent 12 months of GnRHa treatment, 40 treatment-naïve girls with ICPP, and 19 age-matched girls with premature thelarche underwent resting-state functional magnetic resonance imaging using a 3T MRI. VMHC method was performed to explore the differences in the resting-state interhemispheric functional connectivity. The levels of serum pubertal hormones, including luteinizing hormone (LH), follicular-stimulating hormone, and estradiol, were assessed. Correlation analyses among the results of clinical laboratory examinations, neuropsychological scales, and VMHC values of different brain regions were performed with the data of the GnRHa treated group.

**Results:** Significant decreases in VMHC of the lingual, calcarine, superior temporal, and middle frontal gyri were identified in the untreated group, compared with the control group. Medicated patients showed decreased VMHC in the superior temporal gyrus, when compared with the controls. Compared to the unmedicated group, the medicated group showed a significant increase in VMHC in the calcarine and middle occipital gyrus. Moreover, a positive correlation was observed between basal LH levels and VMHC of the middle occipital gyrus in medicated patients.

**Conclusions:** These findings indicate that long-term treatment with GnRHa was associated with increased interhemispheric functional connectivity within several areas responsible for memory and visual process in patients with ICPP. Higher interhemispheric functional connectivity in the middle occipital gyrus was related to higher basal LH production in the girls who underwent treatment. The present study adds to the growing body of research associated with the effects of GnRHa on brain function.

## Introduction

Central precocious puberty (CPP) is defined as the occurrence of secondary sexual characteristics before 8 years of age in girls, due to the premature activation of the hypothalamic-pituitary-gonadal (HPG) axis (1–3). The term “idiopathic CPP” (ICPP) refers to cases without central nervous system lesions, such as hypothalamic tumors or other detected lesions. ICPP accounts for the majority of cases of CPP (4). Gonadotropin releasing hormone analogs (GnRHa) are most commonly and effectively used for the management of CPP. Long-acting GnRHa function by decreasing the release of luteinizing hormone (LH) and follicle-stimulating hormone (FSH), and consequently, the production of sex steroids. GnRHa have wide applications in the management of several gynecological diseases and for treating gender dysphoria (5).

It is thought that puberty represents a second organizational period in brain development, which features dramatic fluctuations in pubertal hormone levels (6, 7). Recent study has reported that alterations in the amplitude of low-frequency fluctuation in the superior temporal gyrus and right superior frontal gyrus in girls with early HPG-axis activation, and higher prolactin (PRL) levels were associated with increased activity in the right superior frontal gyrus (8). Earlier studies have revealed the effects of the premature activation of the HPG axis on brain structure and function (8, 9). However, the influence of GnRHa on the underlying processing of brain networks is still poorly understood, when used as standard hormone suppression treatment for CPP.

To date, only a few studies have explored changes in brain function following GnRHa treatment in humans, but these studies have mainly focused on young women and adolescents with gender dysphoria. For instance, one study reported that cerebral blood flow decreased in the dorsolateral prefrontal cortex and inferior parietal and temporal lobes in young women after GnRHa treatment (10). However, both resting-state fMRI (RS-fMRI) and diffusion tensor imaging studies in adolescents with gender dysphoria (irrespective of whether they were transmen or transwomen) failed to find a significant effect of GnRHa on brain development (11, 12). Prospective trials with pubertal girls are needed to elucidate the effects of GnRHa on brain function development.

Although GnRHa therapy is clearly beneficial in stabilizing pubertal symptoms and improving adult height in patients with CPP (2, 13), studies with young women suggest that GnRHa may have negative effects on memory (14, 15), eliciting concern regarding the potential psychosocial outcomes of GnRHa administration during adolescence (16). To date, evidence regarding the deleterious effects of GnRHa on the cognitive, behavioral, and social functions in patients with CPP has not been found (17, 18).

RS-fMRI is a non-invasive technique for investigating intrinsic brain activation patterns, without a task-related bias. Voxel-mirrored homotopic connectivity (VMHC) is an effective method to evaluate interhemispheric functional connectivity (FC) using fMRI data. Studies on normal development using homotopic RSFC showed region-specific developmental trajectories across the lifespan of the individual (19). Therefore, VMHC is an appropriate and relatively novel approach for exploring the effects of GnRHa on brain function development in girls with CPP.

To best of our knowledge, no study has examined the effects of GnRHa on cognition and brain function with regard to the interhemispheric RSFC changes in patients with CPP. This study examined interhemispheric RSFC in ICPP girls who had not received GnRHa therapy (unmedicated group), those who had been treated with GnRHa for about 12 months (medicated group), and age-matched girls with premature thelarche (control group). The aim of the present study was to investigate changes in VMHC following GnRHa treatment for 12 months in girls with ICPP. We also explored the endocrine mechanism by which GnRHa affects brain function. There is no hypothesis regarding the effect of GnRHa on brain functional connectivity, as it has not been investigated in patients with CPP.

## Methods

### Participants

**Figure 1:**
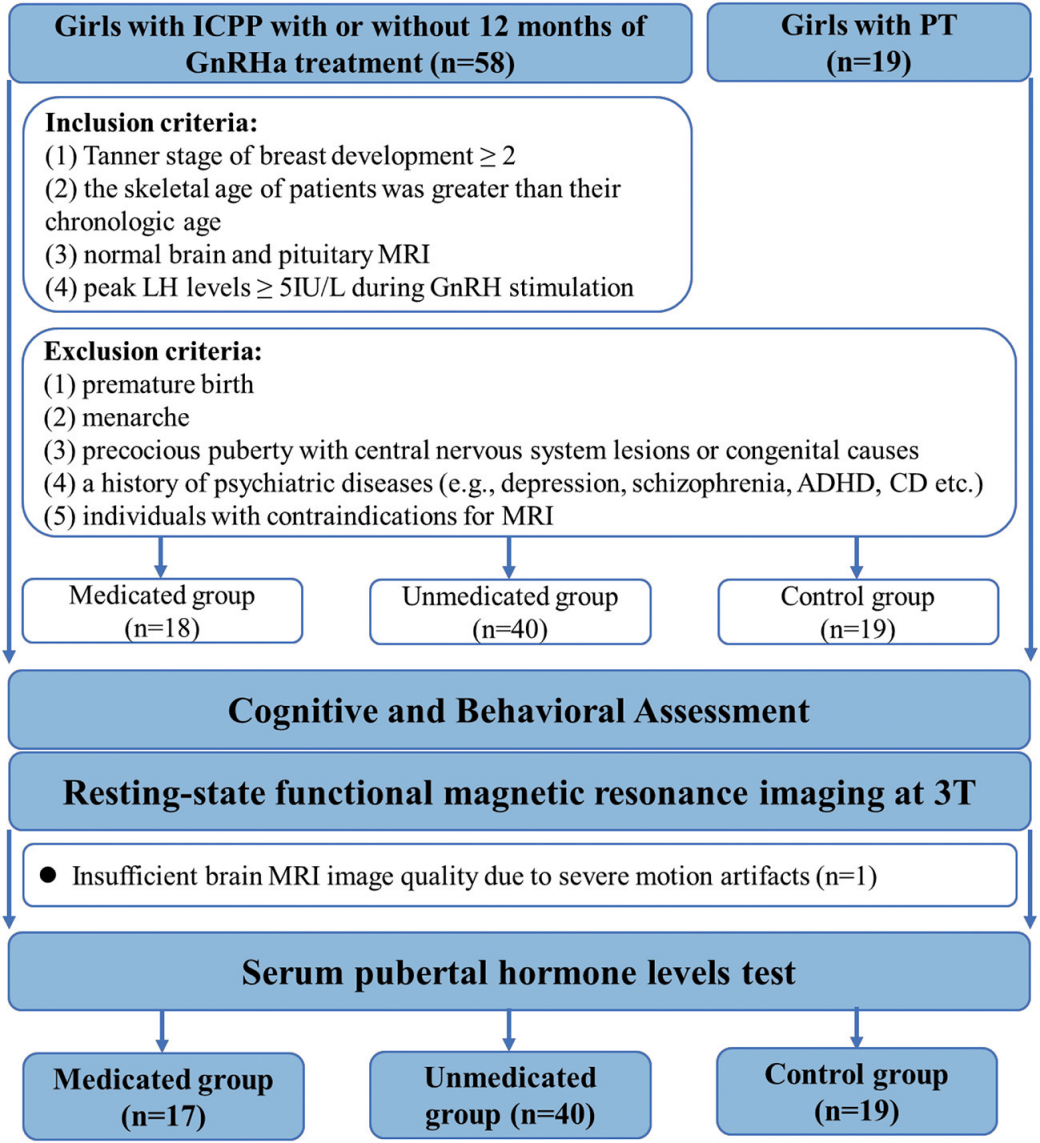
Flowchart for selecting the study population. GnRHa, gonadotropin releasing hormone analogs; LH, luteinizing hormone.

A total of 77 participants were enrolled in this study. Fifty-eight girls diagnosed with ICPP and 19 girls diagnosed with premature thelarche, before the age of 8 years (13 patients had been first diagnosed at other hospitals), were recruited from the Child Healthcare Department of the Second Affiliated Hospital of Wenzhou Medical University. The diagnostic criteria for ICPP included: (1) Tanner stage of breast development ≥ 2, (2) the skeletal age of patients was greater than their chronologic age, (3) normal brain and pituitary MRI, and (4) peak LH levels ≥ 5IU/L during gonadotropin hormone releasing hormone (GnRH) stimulation (2). The exclusion criteria for all groups included: (1) premature birth, (2) menarche, (3) precocious puberty with central nervous system lesions or congenital causes, (4) a history of psychiatric diseases [e.g., depression, schizophrenia, attention deficit hyperactivity disorder (ADHD), conduct disorder (CD) etc.] and (5) individuals with contraindications for MRI (Figure 1).

There is consensus that not all patients with CPP need medical intervention. Medical treatment is indicated when the natural course of the disease is likely to result in adult short stature, early menarche, or severe psychological problems in the affected children; specific treatment to postpone menarche or to reduce psychological stress is not recommended (20, 21). Eventually, in accordance with our departmental policy, the prospect of GnRHa therapy was discussed with the patients and their parents, especially with regard to the risk of adult short stature. Based on acceptance or refusal of GnRHa therapy, all patients were categorized into treated and untreated groups.

Eighteen patients who received GnRHa therapy were categorized into the treated group. The medicated group received triptorelin (3.75 mg) or leuprorelin every 4 weeks, subcutaneously or intramuscularly (mean duration ± standard deviation, 12.71 ± 2.91 months). Treatment regimens were adjusted according to each patient's condition. During the treatment course, the dosage was halved for 2 patients and the type of medication was changed for 2 others.

Forty girls with ICPP who did not receive GnRHa therapy comprised the ICPP group. One reason for refusal of GnRH treatment was the potential side effects such as local erythema, hyperlipidemia, central obesity, temporary vaginal bleeding, osteoporosis, and infertility (22, 23) Another reason for refusal was that the expected adult height was almost equal to the predicted adult height.

Nineteen girls with premature thelarche comprised the control group. Premature thelarche was diagnosed on the basis of isolated breast development and a peak LH level <5 IU/L on GnRH stimulation test (20). Because girls with advanced pubertal stage present increased prevalence of behavioral problems compared to those with normal pubertal stage, we selected girls with isolated, premature thelarche as our “controls,” thereby controlling for the effect of early development of secondary sexual characteristics (7) and isolating the effects of pubertal suppression.

The duration of illness was defined as the number of months between the scan and first diagnosis, which was based on medical records maintained at our hospital or other (previous) hospitals. Radiographs of the left hand and wrist were used to evaluate the skeletal age. The pubertal stage (Tanner scale) of all participants was assessed by inspection and palpation by a pediatric endocrinologist. All patients were pre-menarcheal and were classified as at least Tanner stage 2 of breast development.

The study was approved by the Ethics Committee of the Second Affiliated Hospital of Wenzhou Medical University. We obtained informed written consent from the appointed proxies for all patients. After initial selection, o participant from the medicated group was excluded from further MRI data analysis, due to the presence of several movement artifacts on the image.

### Cognitive and Behavioral Assessment

The full-scale intelligence quotient (IQ) was assessed using the Wechsler Abbreviated Scale of Intelligence for Chinese Children-Revised (WISCC-R). Behavioral assessment was performed using the parent-reported Child Behavior Checklist (CBCL) (24).

### Hormonal Assays and Gonadotropin Hormone Releasing Hormone Stimulation Test

The GnRH stimulation tests were performed at 8:00 a.m. for all patients. Blood samples were evaluated using electrochemiluminescence immunoassay. Serum LH and FSH levels were measured at 0, 30, and 60 min. E2 was measured at 0 and 30 min after gonarelin injection (2.5 μg/kg, maximum dose: 100 μg). The maximum measured levels of serum LH, FSH, and E2 were regarded as the peak LH, FSH, and E2 levels, respectively. Hormone levels measured at 0 min were treated as baselines. Hormone concentrations were expressed according to minimum detection values (assay sensitivity = 0.2 IU/L). A peak LH level ≥ 5 IU/L after GnRH stimulation indicated HPG-axis stimulation (25). A suppressed luteinizing hormone after GnRHa administration (peak LH ≤ 3 IU/L) indicates that the treatment was having the desired effect (23).

### Magnetic Resonance Imaging Acquisition

MRI was performed 1–2 days prior to the GnRH stimulation examination. Structural and functional MRI was performed using a 3.0 T GE Healthcare-Signa HDxt 3T MRI scanner (General Electric, Milwaukee) with an eight-channel phase array head coil. We used noise-reducing headphones and sponge pads to minimize head movement. The high-resolution 3-dimensional structural image data was collected using the whole brain spoiled gradient echo sequence [repetition time = 8.88 ms; echo time = 4.02 ms; inversion time = 900 ms; flip angle = 15°; matrix size =256 × 256; field-of-view = 256 × 256 mm; slice thickness =1 mm, 160 slices]. Participants were instructed to lie quietly and keep their eyes closed for the resting-state functional scan. The images were acquired with a gradient echo-planar imaging sequence (repetition time = 2,500 ms, echo time = 40 ms, flip angle = 90, slice thickness = 4 mm, slice gap = 4 mm, matrix size = 64 × 64, and field-of-view = 256 × 256, 34 slices). The scan time was 6 min.

### Functional Magnetic Resonance Imaging Image Preprocessing

All preprocessing was performed using SPM8 (http://www.fil.ion.ucl.ac.uk/spm) and Data Processing Assistant for RS-fMRI (http://www.restfmri.net) for the RS-fMRI data. The first 10 frames were discarded to account for hemodynamic delay. Preprocessing comprised slice-timing correction for interleaved acquisitions, head motion correction (head motion was <2.5-mm translation in the x, y, or z directions, or <2.5° of angular rotation along the three axes), spatial registration of the high resolution structural T1 images for each participant, and adjustment of the time series of the images by removing the linear drift. Normalized images were smoothed at 6-mm full width, at half maximum of the isotropic Gaussian kernel. Several nuisance signals, including the 6 motion parameters, white matter (WM), and cerebrospinal fluid (CSF) were regressed for temporal correction. Subsequently, the RS images were bandpass filtered between 0.01 and 0.08 Hz to reduce low-frequency drift, and physiological high-frequency respiratory and cardiac noise. Segmentation of the anatomical image into gray matter, WM, and CSF, and normalization of the anatomical and functional images to the standard Montreal Neurological Institute (MNI) brain space (voxel size = 3 mm^3^) were performed.

### Voxel-Mirrored Homotopic Connectivity Analysis

We averaged spatially normalized T1 images of the 76 participants to generate a mean normalized T1 image, to obtain a left-right hemisphere symmetric brain template and its left-right mirror version was used to make the template (26). Subsequently, each individual T1 image was normalized to standard MNI space using the symmetrical template. Pearson's correlation was performed for the time-series of each voxel and that of its symmetrical interhemispheric counterpart, resulting in individual VMHC maps. The VMHC map from each individual participant was transformed into a Z-values map, using Fisher's transformation for group level analysis.

### Statistical Analysis

The comparison analysis for demographic, psychometric measures, and basal pubertal hormone levels was performed using a one-way analysis of variance (ANOVA). The resulting significant measures underwent pairwise *t*-tests (2-tailed) between groups, with the exception of comparisons between the patient groups. The Holm–Bonferroni method was used to correct for multiple comparisons on *post-hoc* pairwise tests. Two-sample *t*-tests were used to compare the peak hormone levels and duration of illness between the medicated and unmedicated groups. Given that the distribution of hormonal data was negatively skewed (Kolmogorov–Smirnov test, *P* < 0.05), the hormonal data underwent logarithmic transformations to improve its normality. The logarithmic transformations of the hormone levels were used in subsequent analyses.

Individual VMHC maps were entered into a voxel-wise one-way ANOVA, to examine the differences in the interhemispheric FC among the groups. Independent samples *t*-tests were performed for significant ANOVA measures, to evaluate pair-wise differences among the three groups. Significant differences in VMHC among the three groups were defined at the voxel level as *P* < 0.001. The cluster threshold was set at > 25 voxels and *P* < 0.05 corrected by AlphaSim multiple comparison correction. Partial correlation analysis was conducted to assess the relationships between the VMHC values (that had significant difference in the structural volume among the different groups) and clinical variables that were different among the different groups by controlling for age during screening. Moreover, a correlation analysis was performed between VMHC and age in each group, to further exclude age from the results. *P* < 0.05 was considered statistically significant. All statistical analyses were performed using SPSS version 25.

You may insert up to 5 heading levels into your manuscript as can be seen in “Styles” tab of this template. These formatting styles are meant as a guide, as long as the heading levels are clear, Frontiers style will be applied during typesetting.

## Results

### Clinical Characteristics and Levels of Hypothalamic-Pituitary-Gonadal Axis Hormones

The demographic characteristics of the participants and clinical information are shown in Table 1. Significant group differences were found in height [*F*_(2, 76)_ = 4.387, *P* = 0.016] and weight [*F*_(2, 76)_ = 4.395, *P* = 0.016]. *Post-hoc* independent-sample *t*-tests demonstrated that participants in the medicated [*t*_36_ = 4.109, *P* = 0.021] and unmedicated [*t*_(59)_ = 4.111, *P* = 0.006] groups were significantly taller, compared to the control group. There were no differences in height between the medicated and unmedicated groups [*t*_47_ = 0.001, *P* = 0.999]. The difference between the medicated and control groups showed a trend toward higher weight [*t*_36_ = 3.571, *P* = 0.053]. Participants in the unmedicated group weighed significantly more than the controls [*t*_59_ = 4.457, *P* = 0.004].

**Table 1:** Demographics and neuropsychological data.

	**Study group**^**a**^		
		**Patient group**^**b**^	
	**Control** **(*n* = 19)**	**Medicated** **(*n* = 17)**	**Unmedicated** **(*n* = 40)**
**Demographic and clinical data**			
Age, y	9.9 (0.5)	10.1 (0.6)	10.0 (0.5)
Height, cm	136.8 (5.1)	140.9 (6.0)	140.9 (5.0)^*^
Weight, kg	31.1 (5.4)	34.7 (5.1)	35.6 (5.6)^*^
BMI	16.5 (2.1)	17.4 (1.8)	17.9 (2.3)^*^
IQ (WASI-CR)	105.6 (11.4)	104.8 (10.8)	106.9 (13.0)
CBCL (total)	14.1 (14.0)	10.3 (11.3)	12.1 (13.6)
**Pubertal hormone data**			
Basal LH, IU/L	0.35 (0.48)	0.41 (0.62)	2.22 (1.71)^**^
Basal FSH, IU/L	3.45 (1.19)	1.63 (1.27)	5.97 (6.07)^**^
Basal E2, IU/L	35.09 (17.19)	21.52 (10.17)	52.80 (27.10)^**^
Peak LH, IU/L	NA	3.01 (7.67)	27.63 (14.32)^**^
Peak FSH, IU/L	NA	3.73 (4.63)	13.91 (5.93)^**^
Peak E2, IU/L	NA	23.88 (10.20)	55.21 (26.53)^**^
Duration of illness, month^c^	NA	37.48 (9.88)	38.47 (9.21)
**GnRHa use**			
Duration, month	NA	12.7 (2.9)	NA
Dose, mg	NA	46.2 (10.8)	NA
**GnRHa type at mri scan, No. of participants**			
Triptorelin	NA	3	NA
Leuprorelin	NA	14	NA

*CBCL, the parent-rated Child Behavior Checklist; E2, estradiol; FSH, follicle-stimulating hormone; GnRHa, gonadotropin releasing hormone analogs; LH, luteinizing hormone; NA, not applicable; WISC-CR, Wechsler Abbreviated Scale of Intelligence for Children-Chinese Revised*.

a *All data is expressed as mean (SD)*.

b *Medicated means girls with ICPP who had been treated for a number of months with gonadotropin releasing hormone analogs; unmedicated means girls with ICPP who were not were received GnRH analog treatment at the time of assessment*.

c *Duration of illness is defined as the number of months between the time of scan and first diagnosis, which was based on medical records from our hospital or other previous hospitals*.

* P < 0.05;

** *P < 0.005*.

Significant group differences in basal LH levels [*F*_(2, 76)_ = 18.800, *P* < 0.001], basal FSH levels [*F*_(2, 76)_ = 6.038, *P* = 0.004], and basal E2 levels [*F*_(2, 76)_ = 12.985, *P* < 0.001] were observed. *Post-hoc* independent-sample *t*-tests demonstrated that the medicated and control groups showed significantly lower basal LH levels compared with those of the unmedicated group (both *P* < 0.001). Basal LH levels did not differ between the controls and medicated (*P* > 0.05). The medicated (*P* < 0.001) and control groups (*P* = 0.016) showed significantly lower basal E2 levels compared with those of the unmedicated group. Basal E2 levels did not differ between the control and medicated groups (*P* > 0.05). The medicated group (*P* = 0.004) showed significantly lower basal FSH levels compared with those of the unmedicated group. Basal FSH levels did not differ between the control and medicated groups, as well as the control and unmedicated groups (both *P* > 0.05). Moreover, two-sample t-tests showed that the medicated showed a significantly lower peak LH level, peak FSH level, and peak E2 level compared with the unmedicated group (both *P* < 0.001). However, no significant group differences were found in age [*F*_(2, 76)_ = 1.099, *P* = 0.339], body-mass index [*F*_(2, 76)_ = 2.450, *P* = 0.093], PRL [*F*_(2, 76)_ = 0.680, *P* = 0.712], or cortisol [*F*_(2, 76)_ = 0.801, *P* = 0.670].

### Interhemispheric Connectivity Differences

**Figure 2:**
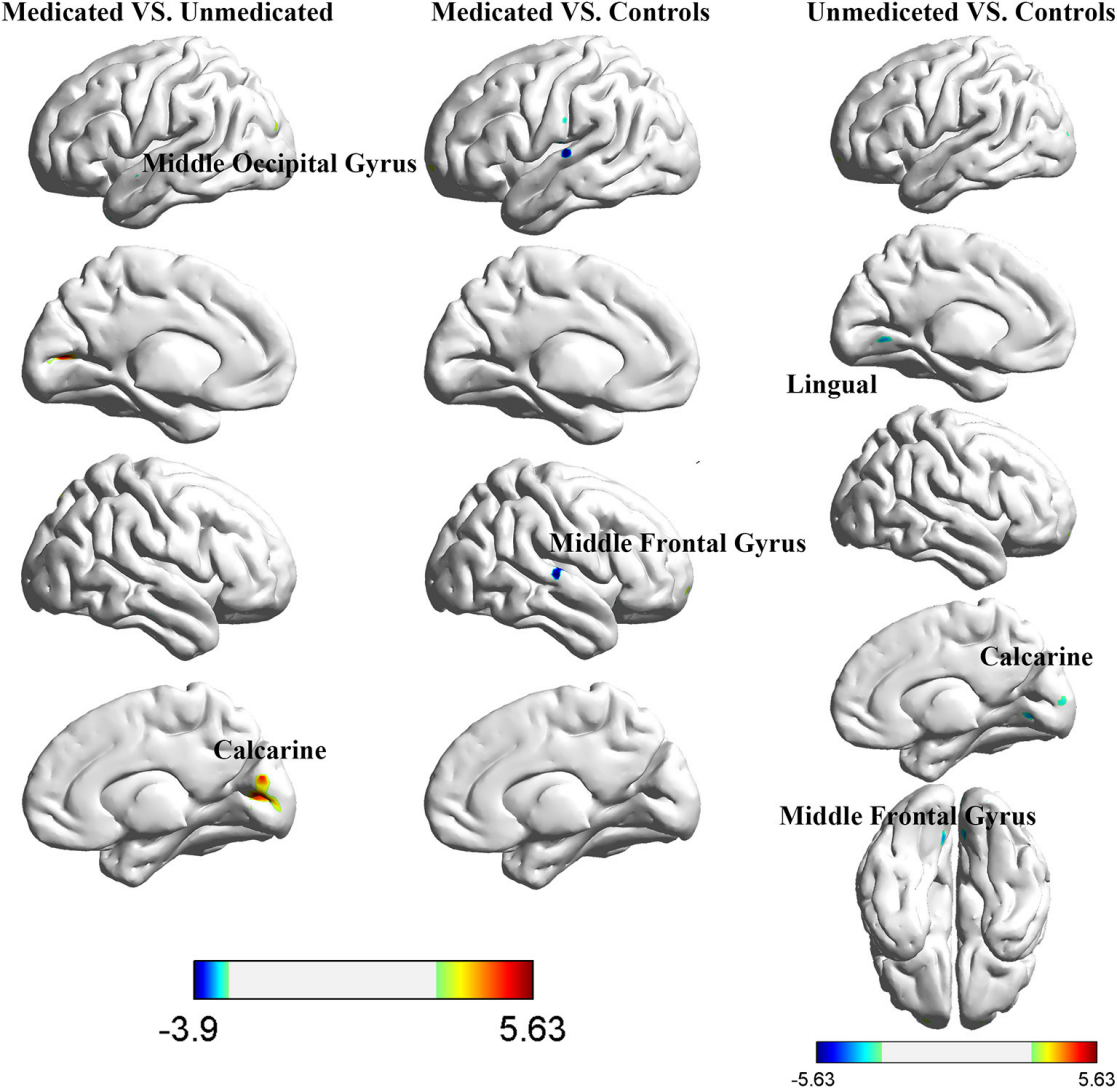
Differences in interhemispheric functional connectivity among healthy control participants and girls with ICPP.

The between-group interhemispheric connectivity comparisons are shown in Table 2 and Figure 2. First, comparison between the control and untreated groups revealed significant reductions in VMHC in the unmedicated group within the lingual gyrus, calcarine gyrus, superior temporal gyrus, and middle frontal gyrus (MFG). No areas of increased VMHC were observed in the unmedicated group, when compared with the control group. Second, compared to the control group, the medicated group showed significant decrease in VMHC in the superior temporal gyrus. VMHC was not higher in any of the regions in the medicated group, when compared to that in the control group. Finally, the medicated group showed a significant increase in VMHC in the calcarine and middle occipital gyrus (MOG) compared with that in the unmedicated group. No significant decreases in VHMC were observed in the medicated group, when compared to the unmedicated group.

**Table 2:** Regions of group differences in voxel-mirrored homotopic connectivity.

**Region**^**a**^	**Brodmann area**	***T*-value**	**Cluster size, mm^**3**^**	**MNI coordinates**		
				**x**	**y**	**z**
**Unmedicated patient group** **<** **control group**						
Lingual	18	−4.3620	41	±6	−66	−3
Calcarine	18	−4.4243	78	±18	−93	9
STG	13	−3.6222	24	±57	−39	15
MFG	10	−4.0389	27	±33	45	15
**Medicated patient group** **<** **control group**						
STG	42	−5.1543	56	±63	−18	9
**Unmedicated patient group** **<** **medicated patient group**						
Calcarine	23	−5.6288	175	±9	−78	9
MOG	19	−3.9338	40	±30	−81	21

*STG, Superior temporal gyrus; MFG, Middle frontal gyrus; MOG, Middle occipital gyrus; MNI: Montreal Neurological Institute*.

a *The control group compared with the unmedicated patient group, the control group compared with the medicated patient group, the medicated group compared with the unmedicated patient group*.

### Correlations Between Pubertal Hormones and Different Brain Regions

**Figure 3:**
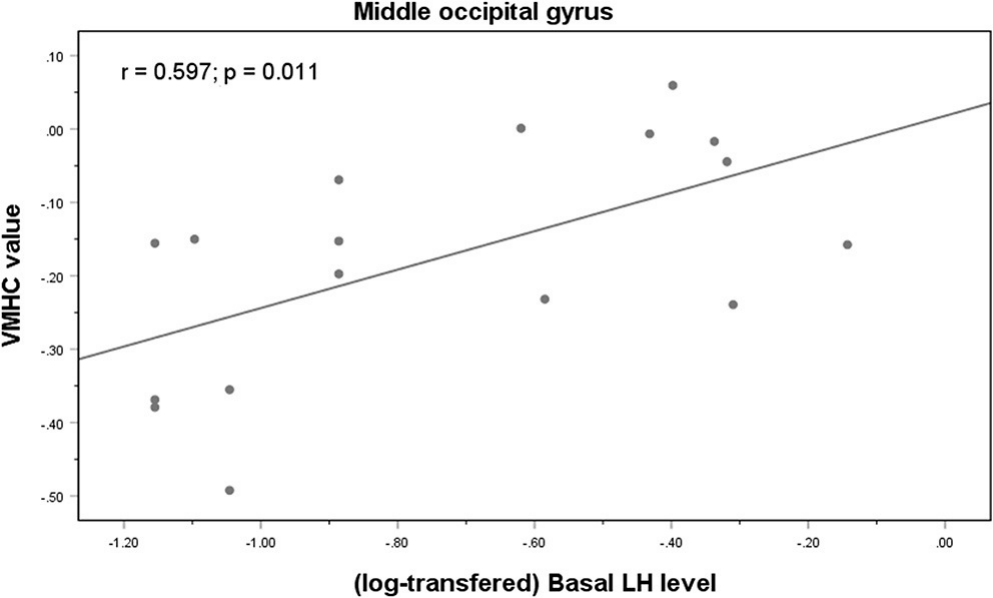
Correlations between VMHC values in the middle occipital gyrus and basal LH levels in the medicated CPP patients.

Correlation analysis revealed a significant positive correlation between the VMHC values in the MOG and the basal LH levels in the medicated group (*r* = 0.597, *p* = 0.011) (Figure 3). It remained significant after controlling for age (*r* = 0.546, *p* = 0.044). In the control group, the VMHC values in the MOG were negatively correlated with both basal (*r* = −0.714, *p* = 0.004) and peak E2 levels (*r* = −0.716, *p* = 0.004), after controlling for the participants' ages during scanning. There was a significant negative correlation between VMHC values in the superior temporal gyrus and basal FSH levels after controlling for age (*r* = −0.365, *p* = 0.026) in the unmedicated group. No significant correlation was observed between VMHC and the CBCL scores in the medicated group (*p* > 0.05).

Additional correlation analyses were performed due to the differences in height and weight among the three groups. Partial correlations analysis was conducted between VMHC values (where there was a significant difference in structural volume among the groups) and clinical variables, which differed among the groups, after controlling for age at scanning, height, and weight. The positive correlation between the VMHC values in the MOG and basal LH levels in the medicated group remained significant (*r* = 0.591, *p* = 0.043). The VMHC values in MOG remained negatively correlated with basal (*r* = −0.774, *p* = 0.003) and peak E2 levels (*r* = −0.755, *p* = 0.005) in the control group.

## Discussion

To the best of our knowledge, this is the first study to demonstrate the effects of long-term GnRHa treatment on brain function in ICPP. Our study had three noteworthy findings. First, both ICPP groups showed significantly reduced VMHC within the superior temporal gyrus compared with that in the control group. Moreover, unmedicated patients also exhibited decreased VMHC within the MFG, MOG, and calcarine gyrus, when compared with the control group. Second, significant increases in VMHC in the calcarine gyrus and MOG were observed in the medicated group compared with the unmedicated group. Third, a significant positive correlation was observed between VMHC in the MOG and basal LH concentrations in the medicated group. Our findings contribute preliminary evidence for the potential endocrine mechanism underlying the effects of GnRHa on brain function in patients with ICPP.

Present study reported the unmedicated patient group showing reduced VMHC in the MFG compared with control group. The MFG is a hub of default mode network (DMN) which is involved in high-order cognitive and emotional behavior regulation (27, 28). Consistent with this findings, our previous structural MRI studies have observed reductions of cortical thickness in the prefrontal cortex of ICPP patients compared to controls (9). Moreover, recent fMRI study has reported altered amplitude of low frequency fluctuation in right frontal gyrus in girls with reactivated HPG axis (8). Decreased interhemispheric connectivity in MFG may suggest intrinsic dysfunctions of the DMN in patients with ICPP.

We observed increased VMHC in the calcarine gyrus and MOG in the medicated group, when compared to the unmedicated group. In addition to visual function (29), primary visual cortex and the occipital gyrus are also responsible for working memory processing (30, 31). Multiple fMRI studies found that visual working memory information can be decoded from early visual cortex during retention (32–34). Makovski et al. explored the role of the visual cortex in retaining visual working memory information using transcranial direct current stimulation reporting that the occipital cortex are involved in working memory consolidation (35). Especially, working memory is one of the main cognitive side effects of GnRHa (36, 37). Increased homotopic connectivity within the occipital cortex which influence the development of girls' working memory in GnRHa treated ICPP may be the crucial neural mechanism of GnRHa's working memory dysfunction.

Previous studies mainly investigated the influence of short-term GnRHa on brain activity in young women, suggesting altered brain activity in the frontal and temporal cortices which belong to default mode network (38–40). There is a possible reason for this inconsistent brain region results, given the differences in duration of treatment and age between the participants of earlier studies and our study. Handa and McGivern (41) posited that visual processing was related to higher-order cognition. Specifically, small deviations in visual processing may have long-term effects on the abstract visualization or conscious recall aspect of higher cognition (42). Thus, the lack of frontal and temporal results in present study may be attributed to the modulation of the visual network prior to default mode network by GnRHa. Besides, the effects of GnRHa on the brain regions of primary systems, including the visual system may become apparent only after long-term treatment (40).

The neuroendocrinological basis of the effect of GnRHa on human brain functional development remains unclear. This preliminary study found a positive correlation between basal LH concentration and MOG VMHC values in the medicated group. Our experimental design made it impossible to differentiation between the direct effects of GnRHa treatment and the secondary effects of LH and FSH suppression and/or E2 secretion. LH and sex steroids could mediate neurogenesis and synaptogenesis (43, 44). These hormones are known to have permanent organization and temporary activation effects on brain maturation (45, 46). Therefore, we speculate that LH might specifically regulate interhemispheric FC by enhancing the neural activity or synaptic coupling in these regions. Our findings can probably be attributed to the indirect effects of GnRHa. GnRHa receptors were recently discovered to be located on neurons within the human brain (47), which provides evidence for the direct effects of GnRHa treatment. Future studies on GnRHa receptors could help to identify the direct and indirect effects of GnRHa and further elucidate the exact mechanism underlying the effects of GnRHa treatment on brain function.

The present study has some limitations. Its design was cross-sectional and thus, considerable differences in interhemispheric connectivity may have existed already between the untreated and treated patients, prior to treatment. There are several reasons, which prove the improbability of these differences. First, the possibility of other diseases affecting brain development was ruled in all participants, who were well-matched for variables such as age, hand preference, IQ, and the course of illness. Second, the relatively long mean duration of GnRHa treatment (12.7 months) made the effect of treatment more significant and credible. Third, the age range of the participants was narrow (9.0–11.4 years) and VMHC values which showed differences among groups were not correlated with age. Thus, it is credible that our results were largely attributable to the treatment. Furthermore, there are existing asymmetries in brain. We tried to mitigate this phenomenon by using a symmetric template and smoothing the functional data. Finally, the sample size used in the present study was relatively small, and future studies with larger sample sizes are required to further substantiate our findings.

This study provided preliminary evidence for the effects of long-term GnRHa therapy on brain function in girls with ICPP. The present study demonstrated that long-term GnRHa therapy is associated with higher interhemispheric connectivity in several areas responsible for memory and visual process and this effect may be associated with LH levels. Longitudinal clinical studies that will compare multimodal neuroimaging (functional and structural MRI) parameters and cognition among GnRHa treated patients with ICPP and age-matched controls with typical development are needed in future to validate the present findings and support the hypothesis that pubertal hormone suppression influences cognition, brain function, and brain structures during the developmental stages.

## Data Availability Statement

The datasets generated for this study are available on request to the corresponding author.

## Ethics Statement

The studies involving human participants were reviewed and approved by The Ethics Committee of the Second Affiliated Hospital of Wenzhou Medical University. Written informed consent to participate in this study was provided by the participants' legal guardian/next of kin.

## Author Contributions

ZY designed and supervised the study. TC, WY, HG, DY, LZ, and XX collected the data. TC and YF collected the participants. TC and WY integrated the data. XL and TC preprocessed and analyzed the data. TC drafted the manuscript. XX, WY, and TC discussed the results and commented on the manuscript.

### Conflict of Interest

The authors declare that the research was conducted in the absence of any commercial or financial relationships that could be construed as a potential conflict of interest.

## Abbreviations

GnRHa: gonadotropin releasing hormone analog
CPP: central precocious puberty
VMHC: voxel-mirrored homotopic connectivity
LH: luteinizing hormone
FSH: follicle-stimulating hormone
PRL: prolactin
E2: estradiol
HPG: hypothalamic pituitary gonadal
FC: functional connectivity
CBCL: parent-rated Child Behavior Checklist
WISC-CR: Wechsler Abbreviated Scale of Intelligence for Children-Chinese Revised
STG: Superior temporal gyrus
MFG: Middle frontal gyrus
MOG: Middle occipital gyrus.
